# Impact of Spatial and Verbal Short-Term Memory Load on Auditory Spatial Attention Gradients

**DOI:** 10.3389/fpsyg.2017.02028

**Published:** 2017-11-23

**Authors:** Edward J. Golob, Jenna Winston, Jeffrey R. Mock

**Affiliations:** ^1^Department of Psychology, University of Texas at San Antonio, San Antonio, TX, United States; ^2^Department of Psychology, Tulane University, New Orleans, LA, United States; ^3^Neuroscience Institute, Tulane University, New Orleans, LA, United States

**Keywords:** working memory, load theory, orienting response

## Abstract

Short-term memory load can impair attentional control, but prior work shows that the extent of the effect ranges from being very general to very specific. One factor for the mixed results may be reliance on point estimates of memory load effects on attention. Here we used auditory attention gradients as an analog measure to map-out the impact of short-term memory load over space. Verbal or spatial information was maintained during an auditory spatial attention task and compared to no-load. Stimuli were presented from five virtual locations in the frontal azimuth plane, and subjects focused on the midline. Reaction times progressively increased for lateral stimuli, indicating an attention gradient. Spatial load further slowed responses at lateral locations, particularly in the left hemispace, but had little effect at midline. Verbal memory load had no (Experiment 1), or a minimal (Experiment 2) influence on reaction times. Spatial and verbal load increased switch costs between memory encoding and attention tasks relative to the no load condition. The findings show that short-term memory influences the distribution of auditory attention over space; and that the specific pattern depends on the type of information in short-term memory.

## Introduction

The auditory system has a special role in attention control because spatial hearing affords panoramic sensitivity that can detect threats, opportunities, and conspecifics at a distance or out of view (Schafer, 1977; Scharf, 1998). Orienting responses show that this acoustic “early warning system” can interrupt ongoing cognitive activities that utilize short-term memory. Interruptions to attend to potentially important information are often beneficial, but extract a cost to ongoing cognitive activities such as maintaining goal-related attentional biases (Cowan, 1995; Braver, 2012) and short-term memory processes (Hughes and Jones, 2001). Nonetheless, even though the onset of a sound is a potent way to induce attention capture, most studies on relations between attention and short-term memory are done using the visual modality.

Loading short-term memory with several items, such as numbers, before trials in a visual attention task increases the likelihood of attention capture by irrelevant singletons (De Fockert, 2013), and has a general effect when the remembered information is not task-relevant (Lavie and De Fockert, 2005). However, effects of short-term memory load on attention can also be selective, with attention capture only when information in short-term memory overlaps with task-relevant information (cf. Logan, 1979; Woodman et al., 2001; Woodman and Luck, 2004; Olivers et al., 2011; Zhao et al., 2011). Short-term memory load may even facilitate performance if the type of information in memory overlaps with distracting information (Kim et al., 2005; Park et al., 2007) cf. (Gil-Gómez de Liaño et al., 2016). There have only been a few studies of short-term memory load on auditory attention capture, and the results are mixed. Berti and Schröger (2003) used an n-back task variant and found that adding a short-term memory load reduced attention capture (Berti and Schröger, 2003). In contrast, Dalton et al. (2009) showed that concurrent short-term memory load during a stimulus-response compatibility task increased attention capture.

Taken together, the specifics of the task, behavioral measures, and potentially stimulus modality are important considerations when drawing conclusions about short term memory influences on attention (Wickens, 2008). Thus, under some conditions short-term memory load induces a more general impairment in attention control, while in others memory load is more selective.

### Rationale for the Current Study

This study will address some limitations of previous behavioral measures by parametrically assessing the influence of short-term memory load on auditory spatial attention. The above studies have all used a point estimate to gauge the influence of memory load on attentional processing, such as the difference in reaction time for compatible vs. incompatible trials or trials with vs. without a salient distractor stimulus. This approach is useful for comparisons between conditions but does not provide insight into how memory load may affect processing within a domain. Parametric studies within a domain are often done to understand how attention affects processing in domains such as space (Cave and Bichot, 1999), frequency (Scharf et al., 1987), faces (Gratton et al., 2013), and lexical associations (McEvoy and Nelson, 1982).

Here we will test the impact of verbal and spatial short-term memory loads on attentional bias over space. The analyses will focus on perceptual-level effects of attention by infrequently shifting a target’s location, where the target response is determined by a non-spatial feature. Thus, the spatial shifts probe the extent that occasionally changing stimulus location affects performance, presumably because a spatial shift captures attention. We chose the spatial domain because attentional benefits decline with distance from an attended location (Eriksen and St. James, 1986; LaBerge and Brown, 1989). These spatial attention gradients are a prime example of capacity limitations and provide a way to analyze the impact of memory load within the spatial domain.

The sustained attention task we used is similar to everyday situations where attention is focused at one location, as when talking or watching television, and an occasional sound is presented at a different location. Sustained attention to one location contrasts with many attention tasks using trial-by-trial cueing of attended location (Mondor and Zatorre, 1995; Rorden and Driver, 2001), and is an important consideration for any differences in results between paradigms. In addition, imaging studies also provide a rationale for making a distinction between sustained attention and cued attention tasks. Two separate attention networks have been associated with either frequent shifts of spatial attention (fronto-parietal network) and sustained attention (cingulo-opercular network) (Dosenbach et al., 2008).

We focus on situations where attention capture is operationalized by the finding that a stimulus feature that is not strictly relevant to performing the task nonetheless affects performance. Concretely, this means that performance will decline when attention is presumably captured, in a bottom–up fashion, by a stimulus that stands out from others by virtue of having a distinct feature, such as a different color (Boot et al., 2005; Lavie and de Fockert, 2006). Another way to quantify the degree of bottom–up attention capture is to have simultaneous presentation of a target and a distractor, with the distractor having an association with a response that is incompatible with the target’s response (de Fockert et al., 2001; Lavie et al., 2004). The extent that incompatible distractors slow reaction time relative to when they are compatible with the target response is used to define the amount of attention capture. A third approach, which will be used in the present study, examines bottom-up attentional capture by having occasional changes in an irrelevant feature dimension within a task-relevant stimulus. Previous work using this strategy found that an occasional change in pitch when discriminating stimuli on the basis of duration slows reaction time (Schroger and Wolff, 1998).

The main hypotheses contrast general vs. specific influences of short-term memory as a function of parametric manipulations of sound location. General theory predicts that under either spatial or verbal short-term memory load reaction time will increase at all locations, with approximately equal magnitude. In contrast, selective load predicts spatial memory load will slow responses at the attentional focus due to overlap with spatial memory, but infrequent lateral shifts would have no effect of load or even faster reaction times. Selective load also predicts a relationship between the location in memory and locations on the attention task, with less slowing of reaction time when the locations overlap. Selective load predicts no verbal load effects because there is no clear overlap with the attention task.

## Materials and Methods

### Participants

A total of 90 young adults were tested (Experiment 1: *n* = 60, age 19.8 ± 2.3 years; M/F = 30/30, 55 right handed; Experiment 2: *n* = 30, age 19.5 ± 2.2 years, M/F = 10/20, 26 right handed). Subjects completed brief surveys indexing musical experience, handedness, and cognitive failures. The musical experience data were collected only in Experiment 1, and will not be reported here as it is part of a separate study. Hearing was screened through self-report of hearing impairments as well as standard audiometric testing (0.5– 8.0 kHz; ≤25 dB), and no subjects were rejected due to hearing issues. Handedness was determined using the Edinburgh handedness inventory (Oldfield, 1971). Two subjects in the spatial group in Experiment 1 were excluded due to low short-term memory accuracy (<60% correct). No subjects were excluded in Experiment 2. Participants gave written informed consent before testing, and the protocol was approved by the Tulane University Institutional Review Board and the University of Texas, San Antonio Institutional Review Board.

### Design and Behavioral Task

Trials in the spatial and verbal load conditions in both experiments had the same format. In load conditions participants heard three stimuli and encoded them into short-term memory. In the spatial load the same stimulus was repeated three times; under verbal load subjects heard three different words. Next, participants were given 10 trials of an attention task, followed by a retrieval probe to verify that the encoded information was retained in memory while performing the attention task. For the no load control in the verbal or spatial conditions all stimuli were the same as the respective memory condition except that the initial three memory items were replaced by three tones. The analysis will focus of the attention task, which was the same in all conditions.

Subjects listened to sounds presented through insert earphones. The stimuli were processed to elicit percepts of having a given sound originate from one of five virtual sound locations (left to right: -90°, -45°, 0° midline, +45° +90°). A choice reaction time task was used to test attention, where on every trial subjects respond based on a non-spatial feature (amplitude modulation rate of white noise). Attentional capture was quantified within the dimension of space by measuring reaction time to different stimulus locations relative to the location where sounds are presented most often (termed “standard location”). By smoothly varying the distance from an attentional focus at the standard location we will define an attention gradient. Our previous work tested the standard location at -90°, 0°, and +90°, and in each instance the fastest reaction times were observed at the standard location (Golob et al., 2017). In the present study the standard location was always at 0°. Analyses then determined if short-term memory load had an influence on the shape of the attention gradient. To assess whether any short-term memory effects are general or specific we compared short-term memory for verbal or spatial information.

Two experiments were conducted, with the primary difference being that Experiment 1 tested separate groups on verbal or spatial load, while Experiment 2 was a within subject comparison. Experiment 1 had a 2 (condition: spatial, verbal) × 2 (load: short-term memory load, no load control) design. In Experiment 2 each subject was given three conditions: no load, spatial load, and verbal load.

### Experiment 1

#### Materials and Apparatus

##### Spatialized sounds

Acoustic stimuli were presented using insert earphones (ER-3A, Etymotic Research, Elk Grove Village, IL, United States). Insert earphones, rather than free-field speakers, were used to minimize potentially confounding effects from indicators of sound sources that can change with visual information or head movements. Virtual sounds were created to generate perceptions of sound sources that originate in the 3-D frontal azimuth plane (left to right: -90°, -45°, 0° midline, +45°, +90°). For each intended location the appropriate interaural time and level differences and head related transfer functions were applied to the sound waveforms (Tucker-Davis Technologies, Gainesville, FL, United States), and were based on the KEMAR model. For each sound the algorithms employed the same cues that are used for sound localization by the auditory system in the natural environment (Middlebrooks and Green, 1991). All stimuli were normalized to a comparable rms level, and prior to the experiment several listeners verified that stimuli had comparable loudness levels. We next describe stimuli that are specific to the spatial and verbal short-term memory items, and then present stimuli used in the attention task.

##### Spatial STM and control condition

For the memory items used in the load condition, five virtual guitar chords (duration = 400 ms) were generated using Sibelius First (version 7.1.3, Avid Technology, Inc.). All chords were major triads in root position separated by a perfect fifth. From lowest to highest, the root pitches were B♭3 (233 Hz), F3 (175 Hz), C4 (262 Hz), G4 (392 Hz), and D5 (587 Hz). Each chord was spatialized at each of the five locations (-90°, -45°, 0°, +45°, +90°) using the methods described above.

For the no-load condition, spatialized pure tones (250 Hz. 400 ms duration, ∼60 dB nHL) were used as placeholders where remembered items were presented and probed during the load condition. The only difference between control and experimental blocks was that subjects were instructed to remember the location of the chords during experimental blocks.

##### Verbal STM condition

In the verbal STM condition three words were presented acoustically from the standard location. Ninety words (45 one syllable, 45 two syllable, 400-650 ms duration, ∼60 dB nHL) were recorded from one male voice. The verbal control condition was the same with spatial load.

##### Attention task

The stimuli in the attention task were white noise (0.1–10 kHz) and lasted 200 ms (5 ms rise/fall times, ∼60 dB nHL). Each stimulus was then amplitude modulated at either 25 or 75 Hz (90% depth). Two amplitude modulation rates provided a non-spatial cue that was easy to discriminate yet also retained the full range of frequencies to optimize sound localization, and also equated the stimulus energy for the 25 and 75 Hz sounds.

### Procedure

Participants were first tested to ensure that they perceived each stimulus near the intended location by marking the perceived location on a sheet of paper relative to an overhead view of head position. They then heard five repetitions of each white noise burst (25 Hz and 75 Hz), followed by a random sequence 25 and 75 Hz stimuli where subjects responded to each amplitude modulation rate with either a left or right hand button press. The mapping of left/right hand to 25/75 Hz was counterbalanced across subjects. The perception of the three stimuli before the attention task and the subsequent probe were similarly tested. Lastly, participants received practice on the trial format by listening to three consecutive tones, then the 10 amplitude modulated white noise, followed by the probe.

All subjects were tested in both the load and no-load conditions for either the spatial or verbal load conditions. Half of the subjects were tested in the spatial short-term memory condition (*n* = 30) and half were tested in the verbal memory condition (*n* = 30). The memory load in the spatial condition consisted of one of the five possible sound locations, while three different words were memorized in the verbal condition. As shown in the results section, these loads had comparable levels of accuracy to the memory probe at the end of each trial. In the spatial load group the no load condition was always first, while the verbal load had the order counterbalanced. The analysis below did not find an effect of fixed vs. counterbalanced order.

In the attention task participants heard a sequence of 10 amplitude modulated sounds, and responded by pressing one of two buttons based on the amplitude modulation rate (25 or 75 Hz) of each sound. Each block required an equal number of right and left responses. The assignment of right and left responses to amplitude modulation rate was the same as in pretesting, and was counterbalanced across subjects. Each block consisted of 150 target detection trials (15, 10-item sequences), where 84% of stimuli were presented at the standard location at midline (0°), and the stimulus onset asynchrony (SOA) was fixed at 1.2 s. Stimuli were presented at each of the other four “shift” locations on 4% of the trials. Participants were asked to respond rapidly but accurately on the basis of the amplitude modulation rate, irrespective of sound location.

The occasional shifts to a new location were used to map the distribution of auditory attention relative to the area of attentional focus (the standard location). Attentional gradients were shown by behavioral measures of median reaction time (for correct trials) and accuracy at each target location across a 180° range.

The last stimulus in a trial was a memory probe to test for accurate retention of the short-term memory item(s) on each trial. In the spatial task the memory probes had the same pitch as the memory item, and 50% of the probes matched the STM item’s location. Similarly, in the verbal task the probe word matched one of the memory items on 50% of the trials (balanced across serial positions). Subjects pressed one of two buttons to indicate whether the probe was a match or mismatch to one of the memory item(s). Right and left hand response assignments to indicate match/mismatch were counterbalanced across subjects.

### Experiment 2

Experiment 2 used the same methods as in Experiment 1, with the following exceptions. First, each subject was tested on both the spatial and verbal load conditions, as well as a no load condition, in counterbalanced order. Thus, statistical analysis of load type was within subjects in Experiment 2 rather than between subjects, as in Experiment 1. Second, in an attempt to make overall memory accuracy comparable for verbal and spatial loads the pool of potential locations for spatial load was reduced to three locations (-90°, 0°, +90°) rather than the five locations used in Experiment 1. Lastly, fewer trials were given in the spatial load condition because in Experiment 1 there were no significant associations between the location in memory and performance on the attention task. The analysis of conjunctions between locations in memory and the attention task required more trials than are needed to define any overall load effects collapsed across memory locations.

#### Data Analysis and Statistics

In Experiment 1 data were analyzed using analysis of variance (ANOVA) tests with a between subject factor of condition (spatial, verbal), and within subject factors of load (no load, load) and stimulus location. Experiment 2 used within subject comparisons of condition (spatial, verbal, no load). Significant effects (*p* < 0.05, two-tailed) involving condition were followed-up by separate analyses using ANOVA within the spatial and verbal conditions. We also tested for differential effects of memory load on stimuli in the left vs. right hemispace. Sequence effects within trials were examined at the standard location as a function of their order (1st, 2nd…10th stimulus in a row). For the spatial load condition an analysis included a factor of item location to determine whether the location of the item in memory influenced any effects of condition, load, or stimulus location. Separate tests were conducted for reaction time and accuracy measures. As presented below, there were large sequence effects involving the first stimulus in the attention task. Consequently, for the main analysis the first stimulus in the group of ten target-detection trials was excluded. Only data from correct memory probe responses were entered into analyses of the attention task. Median reaction time was calculated to limit any influence of occasional outlier reaction times.

## Results

### Experiment 1

#### Short-Term Memory Task Performance

A *t*-test comparing probe accuracy in the verbal and spatial conditions found significantly higher accuracy in the verbal condition [97 ± 1 vs. 85 ± 1%; *t*_(58)_ = 10.2; *p* < 0.001, η = 0.64]. For probe reaction time there was a small but significant difference, with faster reaction times in the verbal condition [1,043 ± 34 vs. 1,162 ± 41 ms; *t*_(58)_ = -2.2; *p* < 0.03, η = 0.08].

#### Short-Term Memory Load × Stimulus Location

**FIGURE 1:**
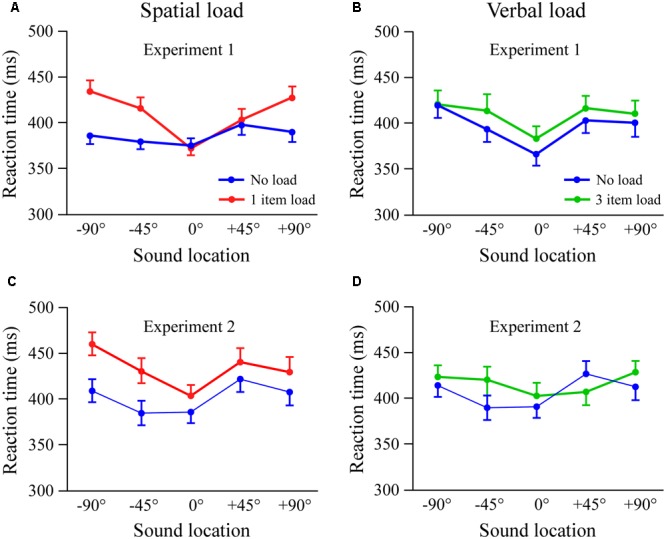
Reaction time as a function of sound location in the spatial **(A,C)** and verbal **(B,D)** load conditions. The first row shows data from Experiment 1 (between subject design), while the second row shows results from Experiment 2 (within subject design). The standard location, where most stimuli originated (*p* = 0.84), is at 0°. Error bars indicate 1 SEM.

Reaction times in the attention task as a function of stimulus location are shown in **Figure 1** for the spatial (**Figure 1A**) and verbal (**Figure 1B**) conditions. The reaction times were calculated based on the 2nd – 10th stimuli in each trial because, as presented below, reaction times to the first stimuli were much longer than the other trials. Reaction time data were analyzed using a two-way repeated measures ANOVA with factors of condition (spatial, verbal), load (no load, load), and stimulus location (5; -90°, -45°, 0°, +45°, +90°).

Analysis of reaction time showed significant effects of condition [*F*_(1,58)_= 7.0; *p* < 0.02, η = 0.11] and location [*F*_(4,232)_= 26.5; *p* < 0.001, η = 0.31], which were qualified by a significant group × condition × location interaction [*F*_(1,58)_= 4.9; *p* < 0.001, η = 0.08]. Follow-up ANOVA tests with factors of load (2) and location (5) were performed in each condition. In the spatial condition there were significant effects of load [*F*_(1,29)_= 7.5; *p* < 0.01, η = 0.21], location [*F*_(4,116)_= 13.4; *p* < 0.001, η = 0.32], and a significant load × location interaction [*F*_(4,116)_= 8.1; *p* < 0.001, η = 0.22]. The load × location interaction indicated that memory load did not affect reaction times at the standard location (mean difference of 2 ms, *p* > 0.80). However, memory load did slow reaction times at the lateral locations relative to the no load condition. In contrast, the verbal condition only had a significant effect of location [*F*_(1,29)_ = 13.9; *p* < 0.001, η = 0.32], which reflected progressively slower reaction times for stimuli away from the standard location. The effect sizes were small for both load (η = 0.04; *p* > 0.25) and load x location (η = 0.02; *p* > 0.65). The potential impact of counterbalancing the load vs. no load blocks in the verbal condition was tested by re-running the verbal analysis but only including subjects that received the no load trials first (*n* = 15). Results also indicated only a significant effect of location [*F*_(4,56)_ = 8.6; *p* < 0.001, η = 0.38]. There was a small overall reaction time difference among no load conditions in the left (*p* < 0.04) but not right hemispace (*p* > 0.60).

Accuracy was examined using a 2 (group) × 2 (load) × 5 (location) ANOVA test. The only significant effect was for location [F_(4,232)_ = 8.8; *p* < 0.001, η = 0.13], with greater accuracy at the midline standard location (96.6 ± 0.4%) vs. lateral locations (range: 92.4% – 93.5%). There was a trend for lower accuracy when under memory load (no load = 94.0 ± 0.7%, load = 93.1 ± 0.6%; *p* < 0.10). Given the lack of accuracy effects for group and load, the analyses below will focus on reaction time, which *a priori* was the most important metric for the hypotheses being tested.

#### Sequence Effects

**FIGURE 2:**
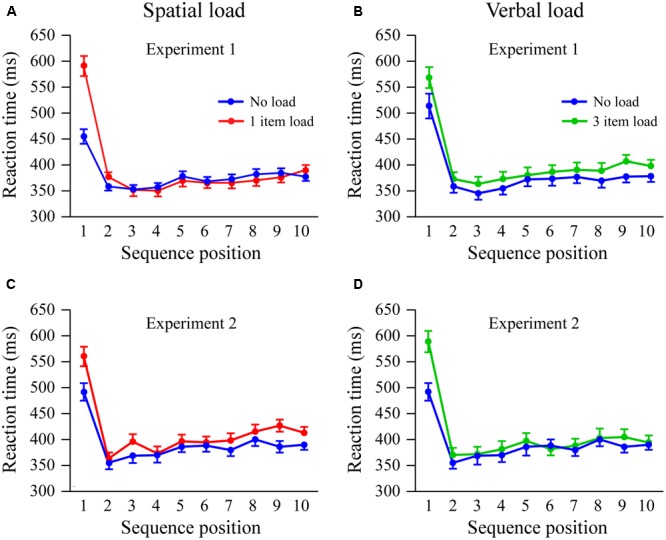
Task switching effects under spatial **(A,C)** and verbal **(B,D)** load. Experiment 1 results are shown in the first row, with Experiment 2 findings in the second row. Sequence indicates serial position of the 10 stimuli in the attention task. Reaction times to the first stimulus after memory encoding were significantly longer relative to subsequent stimuli. Short-term memory load significantly increased reaction times to the first stimulus. Error bars indicate 1 SEM.

In each trial subjects switched from encoding short-term memory item(s) to performing the attention task. As shown in **Figure 2**, reaction times were much longer to the first stimulus in the attention task relative to the following nine stimuli. These sequence effects were evaluated using a condition (2) × load (2) × sequence (10) ANOVA test. There was a significant sequence effect [*F*_(9,522)_= 135.7; *p* < 0.001, η = 0.70], and a small effect of load due to longer reaction times under memory load [*F*_(1,58)_= 6.0; *p* < 0.02, η = 0.09]. There were significant load × sequence [*F*_(9,522)_= 23.7; *p* < 0.001, η = 0.29] and condition × load × sequence interactions [*F*_(9,522)_= 8.6; *p* < 0.001, η = 0.13].

Follow-up ANOVA tests showed that in the spatial condition there was a significant effect of sequence [*F*_(9,261)_= 81.4; *p* < 0.001, η = 0.74] and a load × sequence interaction [*F*_(9,261)_= 31.8; *p* < 0.001, η = 0.52]. Verbal load also had a significant sequence effect [*F*_(9,261)_= 59.6; *p* < 0.001, η = 0.67], but unlike the spatial condition there was only a trend for the load × sequence interaction [*F*_(9,261)_= 2.371; *p* = 0.05, η = 0.08] and a trend toward a load effect [*F*_(1,29)_= 4.1; *p* < 0.06, η = 0.12]. Taken together, load in the spatial condition had a large effect on slowing reaction times when switching task to the first stimulus in the spatial attention task but little effect for subsequent stimuli. Memory load in the verbal condition had a small trend for slowing reaction time, particularly for the first stimulus.

#### Spatial Short-Term Memory Load: Item Location vs. Stimulus Location

The spatial task allowed us to ask whether the location of information in short-term memory had an impact on the reaction time profile in the attention task. A 5 (memory location: -90, -45, 0, +45, +90) × 5 (stimulus location) ANOVA had a main effect of location as described above [*F*_(4,112)_= 13.4; *p* < 0.001, η = 0.32], but no significant effects of memory location or a memory × stimulus location interaction (*p*’s > 0.50, η’s < 0.03).

### Experiment 2

In Experiment 2 subjects performed the same tasks as in Experiment 1 except each subject performed all of the conditions (spatial, verbal, no load). In the spatial condition the pool of memory locations was reduced to three (-90°, 0°, +90°), in an attempt to improve recognition accuracy.

#### Short-Term Memory Task Performance

Memory probe accuracy was significantly higher in the verbal vs. spatial condition [96 ± 1 vs. 86 ± 2%, respectively; *t*_(29)_ = 5.9; *p* < 0.001, η = 0.55]. Reaction time to probes was also faster in the verbal condition [*t*_(29)_ = -3.3; *p* < 0.01, η = 0.27].

#### Short-Term Memory Load × Stimulus Location

Reaction times in the attention task are shown in **Figure 1** for the spatial (**Figure 1C**) and verbal (**Figure 1D**) conditions. As in Experiment 1, reaction times were calculated based on the 2nd – 10th stimuli in the attention task because responses to the first stimulus were much slower than the others. Reaction time data were analyzed using a two-way repeated measures ANOVA with factors of condition (spatial, verbal, no load), and stimulus location (5; -90°, -45°, 0°, +45°, +90°).

There were significant effects of condition [*F*_(2,58)_= 8.2; *p* < 0.01, η = 0.22], location [*F*_(4,116)_= 9.0; *p* < 0.001, η = 0.24], and a condition × location interaction [*F*_(8,232)_= 2.6; *p* < 0.03, η = 0.08]. As above, separate comparisons of verbal and spatial load with the no load condition were conducted. For spatial load there were significant effects of condition [*F*_(1,29)_= 13.5; *p* < 0.001, η = 0.32], location [*F*_(4,116)_= 10.3; *p* < 0.001, η = 0.26], and a weak trend for a condition × location interaction (*p* < 0.11, η = 0.07). A subanalysis of only those subjects with memory probe accuracies > 90% (*n* = 15, spatial = 94.7 ± 1.1%, verbal = 97.1 ± 0.7%) had the same findings (condition *p* < 0.01, η = 0.45; location *p* < 0.01, η = 0.24), with a somewhat stronger condition × location interaction (*p* < 0.08, η = 0.15). These results show that spatial load induced a general slowing of reaction time, but here did not significantly alter the shape of the reaction time profile over locations. Analysis of verbal load revealed a significant effect of location [*F*_(4,116)_= 4.3; *p* < 0.01, η = 0.13] and a small condition × location interaction [*F*_(4,116)_= 3.9; *p* < 0.02, η = 0.12]. The interaction was complex, with a tendency for slowing of reaction times in the left hemispace and an irregular pattern among conditions on the right side.

Analysis of accuracy using a 3 (condition) × 5 (location) ANOVA test had significant effects of condition [*F*_(2,58)_= 6.0; *p* < 0.01, η = 0.17] and location [*F*_(4,116)_= 14.5; *p* < 0.001, η = 0.33]. The condition effect was due to greater accuracy for no load (92.9 ± 0.9%) vs. the load conditions (spatial = 89.7 ± 2.3%; verbal = 89.9 ± 1.2%). As in experiment 1, the location effect indicated greater accuracy at midline (96.6 ± 0.4%) vs. lateral locations (range: 92.4% – 93.5%).

#### Sequence Effects

Plots of reaction time as a function of condition and sequence are shown in **Figure 2**. A 3 (condition) × 10 (sequence) ANOVA showed, as in Experiment 1, significant effects of condition [*F*_(2,58)_= 5.2; *p* < 0.02, η = 0.15], sequence [*F*_(9,261)_= 83.0; *p* < 0.001, η = 0.74], and a condition × sequence interaction [*F*_(18,522)_= 7.2; *p* < 0.001, η = 0.20]. Separate comparisons of the conditions showed that both spatial [*F*_(9,261)_= 6.6; *p* < 0.001, η = 0.19] and verbal load [*F*_(9,261)_= 13.1; *p* < 0.001, η = 0.31] interacted with sequence. Thus, unlike Experiment 1, where spatial but not verbal load interacted with sequence, here reaction times to the first stimulus after encoding under spatial or verbal load were both substantially longer than in the no load condition.

### Combined Analysis of Experiments 1 and 2

The results in Experiments 1 and 2 were generally consistent in showing a dissociation between the impact of spatial and verbal load on performance in the spatial attention task. However they differed in some details, the most important one being that spatial load slowed reaction times at all locations except the standard in Experiment 1. In Experiment 2 the condition x location interaction did not attain significance, and instead there was a main effect of load that covered all five locations. Verbal load had no significant effects in Experiment 1 but had a complex, weak, interaction with location in Experiment 2. To maximize statistical power in defining the effects of spatial and verbal load, we will next combine all subjects in both experiments for separate analyses of spatial and verbal load. Recall that Experiment 1 had a between subject design for load type (spatial, verbal), which necessitates two separate 2 (condition) × 5 (location) ANOVA tests; one for spatial load and one for verbal load. Accuracy is not presented here, as it was not the main focus of the analysis and the findings were comparable among Experiments.

**FIGURE 3:**
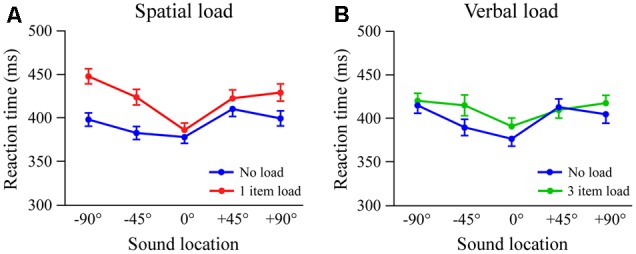
Combined results from Experiments 1 and 2 (*n* = 60). Reaction time as a function of stimulus location in spatial **(A)** and verbal **(B)** load conditions. Error bars indicate 1 SEM.

Results from combining all subjects are shown in **Figure 3**. In the spatial load condition there were main effects of condition [*F*_(1,59)_= 20.4; *p* < 0.001, η = 0.26], location [*F*_(4,236)_= 23.2; *p* < 0.001, η = 0.28], and a condition × location interaction [*F*_(4,116)_= 7.4; *p* < 0.001, η = 0.11] (**Figure 3**). Dividing subjects according to probe accuracy (≥90%, *n* = 21 vs. <90%, *n* = 39) did not affect the results (*p*-values > 0.60). In the verbal condition there was a main effect of location [*F*_(4,236)_ = 15.0; *p* < 0.001, η = 0.20]. Even with a larger number of subjects the effects involving load did not attain significance, although there were trends for condition (*p* < 0.08, η = 0.05) and condition × location (*p* < 0.06, η = 0.04). Overall, spatial load had a marked effect on attention task reaction times, with greater slowing for locations away from the standard. In contrast, verbal load had a minimal effect on reaction times in the spatial attention task.

#### Left vs. Right Shift Locations

We next examine in more detail whether the effects of load at the four lateral locations depend on side (left vs. right hemispace) and eccentricity (±45° vs. ±90°). The statistical analysis combined data from Experiments 1 and 2 using separate 2 (load) × 2 (side) × 2 (eccentricity) × 2 (experiment) ANOVA tests in the spatial and verbal conditions. In the spatial condition there were significant effects of load, [*F*_(1,58)_= 24.9; *p* < 0.001, η = 0.30] and eccentricity [*F*_(1,58)_= 7.0; *p* < 0.01, η = 0.11] indicating slower reaction times with memory load and greater eccentricity. There were also load × side [*F*_(1,58)_= 9.8; *p* < 0.01, η = 0.14] and side × eccentricity [*F*_(1,58)_= 8.3; *p* < 0.01, η = 0.13] interactions, indicating greater load effects on the left side and progressively slower responses with eccentricity on the left but comparable reaction times on the right. The only effect involving experiment was a side x eccentricity interaction [*F*_(1,58)_= 5.4; *p* < 0.03, η = 0.09], that likely reflected slightly different patterns of the means on the right side (see **Figure 1**). In the verbal condition effects of eccentricity and a side × eccentricity interaction were subsumed by a load × side × eccentricity interaction [*F*_(1,58)_= 7.4; *p* < 0.01, η = 0.11] (see **Figure 3**). The three-way interaction reflected slower reaction times under load at -45° on the left but +90° on the right. There were no overall effects of verbal load, and no differences among experiments. Taken together, spatial load had a greater impact on reaction times to shifts on the left side, while verbal load had an irregular pattern of effects across shift locations.

## Discussion

This study examined auditory attention gradients as a function of spatial and verbal short-term memory load. There were four main findings. Relative to no load, a spatial load of one item progressively slowed reaction times for lateral shifts but had little influence on performance at the standard location. In contrast, verbal load did not significantly affect reaction times at any location. In the spatial load condition there was no significant association between the location in memory and performance in the attention task. Lastly, larger switch costs between memory encoding and the first trial of the attention task were found under memory load vs. no load, and did not clearly differ among load conditions. When compared to the initial hypotheses, the results gave some support to both general and specific load theories. We begin by comparing results in the spatial and verbal conditions, and then focus on hypotheses specific to the spatial condition.

### Comparison of Spatial and Verbal Short-Term Memory Load Effects

The dissociation between having load effects for spatial but not verbal information in memory supports specific load theory, as general load predicted slower reaction times under both types of load. The difference between the spatial and verbal load effects is compatible with models having separate types of short-term memory codes that, in turn, would lead to selective load effects depending on the task. Perhaps the best known of these models is Baddeley and Hitch’s working memory theory (Baddeley and Hitch, 1974; Baddeley, 2012), which has separate phonological loop and visuospatial sketchpad stores.

Retention of words in the verbal condition employs the phonological buffer according to Baddeley and Hitch’s working memory model. The combined analysis showed no significant effect of verbal load on reaction time, with no (Experiment 1) or subtle (Experiment 2) verbal load effects in each experiment. The general lack of verbal memory load effects on attention task performance could be due to different coding in short-term memory (phonological) relative to the attention task (non-verbal, spatial). One caveat is that accuracy was greater in the verbal vs. spatial tasks in each experiment, suggesting that the effective load may have been less in the verbal task. It is worth noting, however, that only trials with correct probe recognition were used in the attention task analysis. Subanalysis of high vs. lower performers on probe accuracy did not affect the spatial load findings. Similarly, including only subjects with high levels of probe accuracy under spatial load where probe accuracy differed between spatial and verbal load by only 2.4%, also did not affect the results.

We conclude that accuracy differences in spatial and verbal probe recognition are not likely to account for the different effects of load among verbal and spatial domains. However, additional work would be useful to further test this conclusion, such as testing verbal loads > 3 items, and to get a deeper understanding of the mechanisms for how memory load impacts auditory spatial attention. The attempt to make the spatial task easier in Experiment 2 by having only three potential memory items at cardinal locations separated by 90° was unsuccessful, as performance was numerically improved by only 1%. This provides more evidence that subjects were unlikely to be using a verbal code (e.g., “left,” “middle,” “right” location) to maintain the item location in the spatial task, as accuracy would then be expected to be comparable or greater than the verbal memory task with three items. The potential use of a chunking strategy in the verbal condition could also be examined. Future work may want to explore proactive interference in load effects, since the smaller number of potential items in the spatial task would likely lead to greater proactive interference. Similarly, it may be interesting to consider loads of one vs. >one item because recency effects for the last (only) item are an important factor in probe recognition (McElree and Dosher, 1989; Golob and Starr, 2004). One would expect a greater number of items to have additional negative effects on performance relative to having a single item in memory, which was not seen here, but there may be additional complexities worth exploring. In general, the strong domain (spatial vs. verbal) differences in probe accuracy suggest that what makes the spatial task “difficult” is the use of spatial information in both the memory and attention tasks (Wickens, 2008).

Storage of auditory spatial information is less clear than for words because there has been little work to integrate retention of auditory spatial information with broader working memory models. As the name suggests, the visuospatial sketchpad stores information about visual objects, their locations, and movements for short periods of time (Baddeley, 2012). Prior work shows that dissociations are possible between visual and spatial aspects of this store (Klauer and Zhao, 2004), and that retaining non-verbal information in other modalities might also be included (Smyth and Pendleton, 1990; Bruyer and Scailquin, 1998; Seaborn et al., 2010). We tentatively suggest that short-term memory of auditory spatial information may be retained by the spatial component of the visuospatial sketchpad. The possibility that spatial short-term memory items were recoded into an exclusively verbal or phonological code (e.g., -90° location remembered as “far left,” 0° as “middle,” etc.) was not supported by the findings. If the spatial memory information was recoded into a verbal format then verbal and spatial loads would have the same effect (or lack of effect) on attention task performance. Instead, both experiments showed dissociations between spatial and verbal load effects.

### Memory Load Effects on Attention Gradients

When the results within the spatial condition were examined, none of the three predictions made by specific load hypotheses were strongly supported (i.e., slowing at the standard location, no load effects or faster responses at lateral locations, an association between memory and stimulus locations). The reaction times were slower under spatial memory load at four out of five locations in Experiment 1 and overall slowing was seen in Experiment 2, which gives some support to general load theory. We favor a general load explanation for findings in the spatial condition because the predictions of general load were mostly supported while none of the predictions of specific load theory were correct.

The anomaly for general load is the clear absence of a load effect at the standard location in Experiment 1 and greater slowing at lateral locations vs. the standard in the analysis combining both experiments. We speculate that a general load effect driving slower reaction times at the standard may have been partially counteracted by contextual support in the form of having most stimuli originate at the standard location. Being *ad hoc*, this explanation is not entirely satisfying and would need to be tested more directly in the future. Spatial load also had a greater effect in the left hemispace, which was consistent with early ideas of spatial functions related to the right hemisphere of the brain affecting contralateral spatial processing (Kinsbourne, 1970). Although the current findings lend support to Kinsbourne’s position, one attempt to replicate Kinsbourne’s early short-term memory load results was unsuccessful (Boles, 1979).

Note that although spatial information is not a criterion for which button to press (the non-spatial AM rate is the criterion), spatial information is helpful as a means of using spatial attention to influence stimulus processing on most trials. Many previous studies have shown performance benefits of spatial cueing on tasks where responses are selected on the basis of non-spatial information (Posner, 1978). The v-shaped reaction time curves, with the fastest responses at the 0° standard, show that such benefits exist in our task.

Comparisons between the location of the memory item and the location of stimuli in the attention task did not find any systematic effects on the spatial profile of reaction times. This suggests that although the location of the item was retained in short-term memory, as verified by correct probe responses, there was no additional bias when a stimulus in the attention task was delivered at the memorized location. Prior work in the visual modality has observed faster reaction times to targets in an attention task when they are given at the location in memory, relative to other locations (Awh et al., 1998; Soto et al., 2008). Selective load effects are well-supported, with the impact on visual search depending on the type of items retained in short-term memory (Logan, 1979; Woodman et al., 2001; Woodman and Luck, 2004, 2007), as well as strategic factors (Carlisle and Woodman, 2011). We note that the current findings differ from this prior work not only by being in the auditory modality, but also by the nature of the task. Most of the above studies used visual search tasks, where a set of items are presented together and the subject searches for a target. In the current task items were presented sequentially, which could relate to why there was no clear influence of the memorized location on attention task performance. Given the strength of findings in the visual modality that items in short-term memory influence spatial attention, we believe that the possibility of similar effects in the auditory modality warrants closer examination.

### Short-Term Memory Load and Task Switching

Our protocol necessarily included a task switch between encoding memory items and the beginning of the attention task. There was also a switch to probe recognition, which was of interest just to verify short-term memory storage. Both experiments found that relative to no load, verbal and spatial loads had much slower reaction times when switching from memory encoding to the first of ten stimuli in each trial-block. Such switch costs are common, even when expected by well-practiced subjects (Rogers and Monsell, 1995). Experiment 1 had a significant difference in switch costs among load types (spatial > verbal), but this was not replicated in Experiment 2. Results in Experiment 1 were likely driven by fast reaction times in the control group as opposed to having clear differences among the load conditions.

## Author Contributions

EG and JM contributed to the study design. JW and JM collected the data. EG, JW, and JM analyzed the data. EG and JM wrote the manuscript.

## Conflict of Interest Statement

The authors declare that the research was conducted in the absence of any commercial or financial relationships that could be construed as a potential conflict of interest.
